# Synonymous Variants of Uncertain Silence

**DOI:** 10.3390/ijms241310556

**Published:** 2023-06-23

**Authors:** Christopher J. Giacoletto, Jerome I. Rotter, Wayne W. Grody, Martin R. Schiller

**Affiliations:** 1 Nevada Institute of Personalized Medicine, University of Nevada Las Vegas, 4505 S. Maryland Parkway, Las Vegas, NV 89154, USA; cjg9101112@gmail.com; 2 Heligenics Inc., 10530 Discovery Drive, Las Vegas, NV 89135, USA; 3 School of Life Sciences, University of Nevada Las Vegas, 4505 S. Maryland Parkway, Las Vegas, NV 89154, USA; 4 The Institute for Translational Genomics and Population Sciences, Department of Pediatrics, The Lundquist Institute for Biomedical Innovation at Harbor-UCLA Medical Center, Torrance, CA 90502, USA; 5 Department of Pathology and Laboratory Medicine, University of California Los Angeles School of Medicine, 10833 Le Conte Ave., 705, Los Angeles, CA 90095, USA; wgrody@mednet.ucla.edu; 6 Department of Pediatrics, University of California Los Angeles School of Medicine, 10833 Le Conte Ave., Los Angeles, CA 90095, USA; 7 Department of Human Genetics, University of California Los Angeles School of Medicine, 10833 Le Conte Ave., Los Angeles, CA 90095, USA

**Keywords:** synonymous variant, silent variant, sequence, variant interpretation

## Abstract

Synonymous variants, traditionally regarded as silent mutations due to their lack of impact on protein sequence, structure and function, have been the subject of increasing scrutiny. This commentary explores the emerging evidence challenging the notion of synonymous variants as functionally inert. Analysis of the activity of 70 synonymous variants in the HIV Tat transcription factor revealed that 50% of the variants exhibited significant deviations from wild-type activity. Our analysis supports previous work and raises important questions about the broader impact of non-silent synonymous variants in human genes. Considering the potential functional implications, the authors propose classifying such variants as “synonymous variants of uncertain silence” (sVUS), highlighting the need for cautious interpretation and further investigations in clinical and genetic testing settings.

## 1. Introduction

The genetic code, one of the most important scientific insights of all time, was discovered by Mattheai and Nirenberg and a team of scientists that joined forces [1]. The code is highly redundant, with some variants producing different codons that encode the same reference amino acid. These are referred to as synonymous variants, in contrast to non-synonymous variants that alter codons, changing the encoded amino acid. Synonymous variants are generally considered to be silent for any effect, because they do not change the amino acid sequence of a protein, nor, presumably, its 3D structure [2].

For the past decade, there has been a growing cadre of investigations pursuing unexpected observations in which synonymous variants are associated with a disease, exert selective pressure in fitness assays, or have an effect on protein expression and function (see reviews [3,4,5,6,7,8,9,10,11,12]). Thus, it is becoming more important to understand if these types of variants are rare occurrences, or if they are more commonplace than previously appreciated.

The importance of this problem is well beyond that of a basic science question, as interpretation of genetic sequencing tests generally assumes that synonymous variants are silent. This interpretation is routinely applied for diagnosis and treatment decisions, especially in the modern era of clinical exome and genome sequencing using next-generation sequencing (NGS) methods. Sometimes synonymous variants are referred to as silent variants without conclusive evidence of silence because of the general acceptance of this interpretation, e.g., [13].

In 2022, Shen et al., reported the first estimate of a genome-wide rate of deleterious synonymous variants measuring yeast fitness for a sampling of ~8500 variants in 21 genes [14]. Approximately 76% of variants were not silent, challenging the long-standing assumption of the general silence or benign nature of synonymous variants. Since this publication, three groups have examined and discussed the limitations of this investigation [15,16,17].

Kruglyak et al., suggests that different genetic backgrounds arise from off-target editing in the edited mutant yeast strains and can produce apparent deleterious effects of synonymous variants [17]. Off-target editing is a concern with CRISPR/Cas9 and would be expected to produce different genetic backgrounds for each mutant. While a control could have addressed this possibility, it is not clear how, or how likely it is, that these different genetic backgrounds would affect the overall conclusions of yeast fitness experiments given the number of mutants studied and size of the effect [14].

Dhindsa et al., suggested that there is not enough evidence and provide a supporting analysis on large human genomic datasets indicating that there is little purifying selection of synonymous variants [16]. We suggest that both results are possible and that differences in experiments carried out in the haploid genome of yeast vs. the diploid genome of humans can explain this discrepancy [16]. The yeast fitness experiment was conducted in haploid cells, and it is much easier to isolate and detect the effects of synonymous variants in the absence of wild-type alleles, which may be why they observed 75% of mutants affecting yeast fitness [14]. In human constitutive diseases, most effects of synonymous variants, analogous to the rate observed in the yeast experiments, would only be expressed in a compound homozygous or compound heterozygous recessive mode of inheritance, requiring two deleterious alleles, which would be an infrequent occurrence. Therefore, one would not expect to detect a high frequency of apparent synonymous variants in humans, as only those deleterious synonymous variants paired with another deleterious allele would likely produce an effect. This could explain the apparent paucity of pathogenic entries for such variants in ClinVar and other human disease databases.

In our prior research, we introduced a novel system known as the GigaAssay [18,19], which we have since employed to explore synonymous variants. Specifically, we focused on the HIV Tat protein, a transcription factor responsible for activating the long terminal repeat (LTR) promoter and facilitating HIV gene expression in human cells. Using GigaAssays, we previously examined the impact of saturating single-substitution mutagenesis on the activity of the Tat protein [18,19]. We generated a comprehensive library of Tat variants through saturating mutagenesis, encompassing all 1615 possible single amino acid substitutions. This library was subsequently integrated into a DNA cassette that included the HIV LTR promoter driving the Green Fluorescent Protein (GFP) gene. The initial implementation of the GigaAssay system yielded an exceptionally comprehensive functional map of the Tat protein, facilitating an in-depth examination of structure–activity relationships (SARs). We leveraged this system to investigate synonymous variants.

In close agreement with the Shen et al., paper, we recently determined that 67% of synonymous variants (n = 89) were not silent when the transcriptional activity of the HIV Tat transcription factor was measured with an LTR-GFP reporter [20]. Our paper is based on a standardized measurement of molecular activities of variants in a single-pot multiplexed assay of 1000s of variants at once. Our proprietary GigaAssay^®^ has a demonstrated accuracy of ~95%, as assessed with several methods, and thus is suitable for determining the frequency of non-silent synonymous variants [18,19].

The latter paper adds new important information to the evolving story of non-silent synonymous variants. Our system and design of the approach were advantageous in that it isolates the synonymous variant effect on the direct gene output, drastically reducing the number of potential confounding variables prevalent in examining whole-cell or -organism outcomes such as fitness that are far downstream of its direct function [18,19].

Other advantages of our approach are that the experiment is in human cells, rather than a model organism. However, a noted limitation is that Tat is an exogenous viral gene and may not be representative of human genes. One last advantage is that the GigaAssay is an approach that measures all variants, including those with wild-type and loss of function activities, whereas fitness screens do not measure the variants that are not selected, but rather infer that they are not present because of organism fitness.

## 2. Results

We previously reported a global statistical measure of non-silent synonymous variants for Tat-driven transcription of an LTR-GFP reporter, supporting the conclusion that many synonymous variants in Tat were not silent. Here, each variant was analyzed using the mixed logistic regression model [18,19]. q-values were used to determine if the activity reported was significantly different from that of the wild type (Table S1). Mutants’ activities were compared to the WT activity for all q-values below 0.05. Activities greater than that of the wild type are labeled gain of function (GOF), while activities lower than that of the wild type are labeled loss of function (LOF).

Of the 70 synonymous variants tested, the null hypothesis (activity = WT activity) was rejected in 50% of the variants (n = 35; q < 0.05; Table S1). These results were repeated between two separate cell lines with a 61.3% concordance, and in four cases there were multiple synonymous substitutions for a position that had the same effect on activity. The apparently random sampling presumably arose from random oligonucleotide synthesis errors [20].

While the frequency of non-silent synonymous variants (50%; 35 of 70 demultiplexed by cell line) was lower than the 67% previously reported, the mixed model regression is a more rigorous statistical test, and the conclusion does not change. Our results are also consistent with the observation of non-silent effects of synonymous variants in HIV and other viruses, but our study had an advantage in that it examined the direct variant effect on a gene function such as Tat-driven transcriptional activity, which was not previously assessed [21,22,23,24].

**Figure 1:**
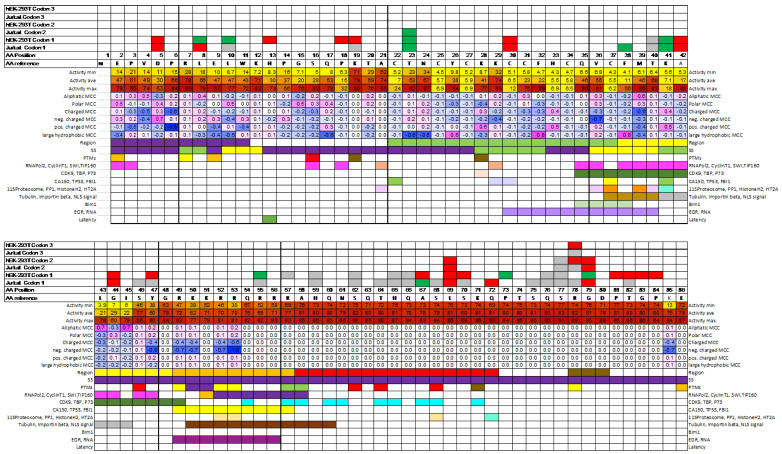
Map of synonymous variants in Tat with their effect on transcriptional function. Each reference amino acid in Tat has from 0 to 5 synonymous substitutions in each codon due to redundancy in the genetic code. hEK-293T and Jurkat cells were analyzed, and a maximum of three different codons were randomly sampled for each position. The map shows the effect of the synonymous variants (codons 1-3) on Tat transcriptional activity at each position for each cell line. Key: loss of function = red; gain of function = green; wild-type activity = grey; no data = white; AA = amino acid, SS = secondary structure, PTM = post-translational modification. The map does not include 10 variants that produced different results among technical replicates or cell lines and are considered indeterminate (also colored white). Minimum, maximum, and average transcriptional activities for the 19 amino acid substitutions at each position are shown as a gradient heatmap, with red indicating WT activity and yellow indicating LOF activity. Matthew’s correlation coefficient (MCC) was used to indicate specificity of each amino acid position for physiochemical characteristics. A gradient shows scores ranging from −1 (blue) to 0 (white) to 1 (magenta). White indicates no specificity, magenta indicates high specificity for the physiochemical group, and blue indicates a high specificity for negative preference against the physiochemical group. Several protein-protein interactions are shown with separate coloring as previously defined [18,19].

We re-analyzed the new synonymous variant classification to assess the effect upon activity. If synonymous variants effects are at the expression or mRNA level, these variants would not be expected to affect, or be correlated with structure, function, or tolerance of amino acids at each position. In general, this was observed, as there were no distinct patterns in secondary, tertiary structure, post-translational modification sites, protein–protein interaction sites, or physiochemical tolerance for each position (Figure 1 and Figure S1).

If the effect on activity were at the mRNA level, one possibility would be contiguous patterns of variants of different effect types, as was observed. WT variants were concentrated in the C-terminal region from amino acids 46 to 86, in which missense variants generally did not affect activity. Both LOF and GOF variants were observed to be clustered in this region. Synonymous variants between 57 and 66 and 75 and 77 retained WT activity, often being replicated in both cell lines (Figure 1). Synonymous variants with LOF activity were also clustered at positions 18–19, 42–47, 67–72, and 78–84. Synonymous variants with GOF activity were randomly distributed throughout the protein.

## 3. Discussion

More investigation will be required to conclusively determine how common non-silent synonymous variants are in the human genome. All of the assays and systems used to measure synonymous variant effects have their own limitations. Our analysis is intriguing but cannot be used by itself to guide interpretation of human variants since Tat is an exogenous viral gene introduced by a lentiviral transduction. Furthermore, the transcriptional reporter measurement is based on haploid Tat, like that in the Shen et al., analysis of the yeast experiments with haploid cells [14].

We suggest several types of investigations going forward. Saturating mutagenesis of synonymous variants in whole-gene studies should be used to directly test the function of a gene in live human cells, as in our Tat study, to minimize confounding variables. More convincing results are needed to better understand the percentage of variants for each potential mechanism. In our work on Tat, the mechanism causing 75% of synonymous variants to not be silent was not determined, with the remaining 25% confined to the first loop in the protein, implicating an effect upon folding that translates to reduced, or complete loss of activity.

Nevertheless, until the variant effect of synonymous variants is better resolved in human systems, clinicians and molecular genetic testing laboratories should consider that synonymous variants may not be silent and perhaps, in appropriate situations, classify them as synonymous variants of uncertain silence (sVUSs). If there is some measure of activity or phenotype that demonstrates that the variant is not distinguishable from the wild type, only then can it be considered truly silent.

## Data Availability

All data are shared as part of this paper.

## Supplementary Materials

The following supporting information can be downloaded at: https://www.mdpi.com/article/10.3390/ijms241310556/s1.

## References

[1] Nirenberg M.W., Matthaei J.H. (1961). The Dependence of Cell-Free Protein Synthesis in *E. coli* upon Naturally Occurring or Synthetic Polyribonucleotides. Proc. Natl. Acad. Sci. USA.

[2] Kimura M. (1977). Preponderance of Synonymous Changes as Evidence for the Neutral Theory of Molecular Evolution. Nature.

[3] Bailey S.F., Alonso Morales L.A., Kassen R. (2021). Effects of Synonymous Mutations beyond Codon Bias: The Evidence for Adaptive Synonymous Substitutions from Microbial Evolution Experiments. Genome Biol. Evol..

[4] Bin Y., Wang X., Zhao L., Wen P., Xia J. (2019). An Analysis of Mutational Signatures of Synonymous Mutations across 15 Cancer Types. BMC Med. Genet..

[5] Hunt R.C., Simhadri V.L., Iandoli M., Sauna Z.E., Kimchi-Sarfaty C. (2014). Exposing Synonymous Mutations. Trends Genet..

[6] Liu Y., Yang Q., Zhao F. (2021). Synonymous but Not Silent: The Codon Usage Code for Gene Expression and Protein Folding. Annu. Rev. Biochem..

[7] Mankodi A., Ashizawa T. (2003). Echo of Silence: Silent Mutations, RNA Splicing, and Neuromuscular Diseases. Neurology.

[8] Sauna Z.E., Kimchi-Sarfaty C. (2011). Understanding the Contribution of Synonymous Mutations to Human Disease. Nat. Rev. Genet..

[9] Seton-Rogers S. (2022). Silent Mutations Make Noise. Nat. Rev. Cancer.

[10] Shabalina S.A., Spiridonov N.A., Kashina A. (2013). Sounds of Silence: Synonymous Nucleotides as a Key to Biological Regulation and Complexity. Nucleic Acids Res..

[11] Lehner B., Crombie C., Tischler J., Fortunato A., Fraser A.G. (2006). Systematic Mapping of Genetic Interactions in Caenorhabditis Elegans Identifies Common Modifiers of Diverse Signaling Pathways. Nat. Genet..

[12] Walsh I.M., Bowman M.A., Soto Santarriaga I.F., Rodriguez A., Clark P.L. (2020). Synonymous Codon Substitutions Perturb Cotranslational Protein Folding in Vivo and Impair Cell Fitness. Proc. Natl. Acad. Sci. USA.

[13] Wang Z., Xu Y., Sun Y., Wang S., Dong M. (2022). Novel Homozygous Silent Mutation of ITGB3 Gene Caused Glanzmann Thrombasthenia. Front. Pediatr..

[14] Shen X., Song S., Li C., Zhang J. (2022). Synonymous Mutations in Representative Yeast Genes Are Mostly Strongly Non-Neutral. Nature.

[15] Mahadevan S. (2022). Silence of the Mutations. J. Biosci..

[16] Dhindsa R.S., Wang Q., Vitsios D., Burren O.S., Hu F., DiCarlo J.E., Kruglyak L., MacArthur D.G., Hurles M.E., Petrovski S. (2022). A Minimal Role for Synonymous Variation in Human Disease. Am. J. Hum. Genet..

[17] Kruglyak L., Beyer A., Bloom J.S., Grossbach J., Lieberman T.D., Mancuso C.P., Rich M.S., Sherlock G., Kaplan C.D. (2023). Insufficient evidence for non-neutrality of synonymous mutations. Nature.

[18] Giacoletto C.J., Schiller M.R. (2023). The History and Conceptual Framework of Assays and Screens. BioEssays.

[19] Benjamin R., Giacoletto C.J., FitzHugh Z.T., Eames D., Buczek L., Wu X., Newsome J., Han M.V., Pearson T., Wei Z. (2022). GigaAssay—An Adaptable High-Throughput Saturation Mutagenesis Assay Platform. Genomics.

[20] Giacoletto C.J., Benjamin R., Deng H.-W., Rotter J.I., Schiller M.R. (2023). Most Synonymous Allelic Variants in HIV Tat Are Not Silent. Genomics.

[21] Cuevas J.M., Domingo-Calap P., Sanjuán R. (2012). The Fitness Effects of Synonymous Mutations in DNA and RNA Viruses. Mol. Biol. Evol..

[22] Jordan-Paiz A., Franco S., Martínez M.A. (2021). Impact of Synonymous Genome Recoding on the HIV Life Cycle. Front. Microbiol..

[23] Wang Q., Barr I., Guo F., Lee C. (2008). Evidence of a Novel RNA Secondary Structurein the Coding Region of HIV-1 Pol Gene. RNA.

[24] Takata M.A., Soll S.J., Emery A., Blanco-Melo D., Swanstrom R., Bieniasz P.D. (2018). Global Synonymous Mutagenesis Identifies Cis-Acting RNA Elements That Regulate HIV-1 Splicing and Replication. PLoS Pathog..

