# Supplementary material for: Synonymous Variants of Uncertain Silence
Source: Int J Mol Sci. 2023 Jun 23;24(13):10556. doi: 10.3390/ijms241310556 (PMC10341574; doi:10.3390/ijms241310556)
Supplement: Supplementary file 1 [file ijms-24-10556-s001.zip › Figure-S1.pdf]

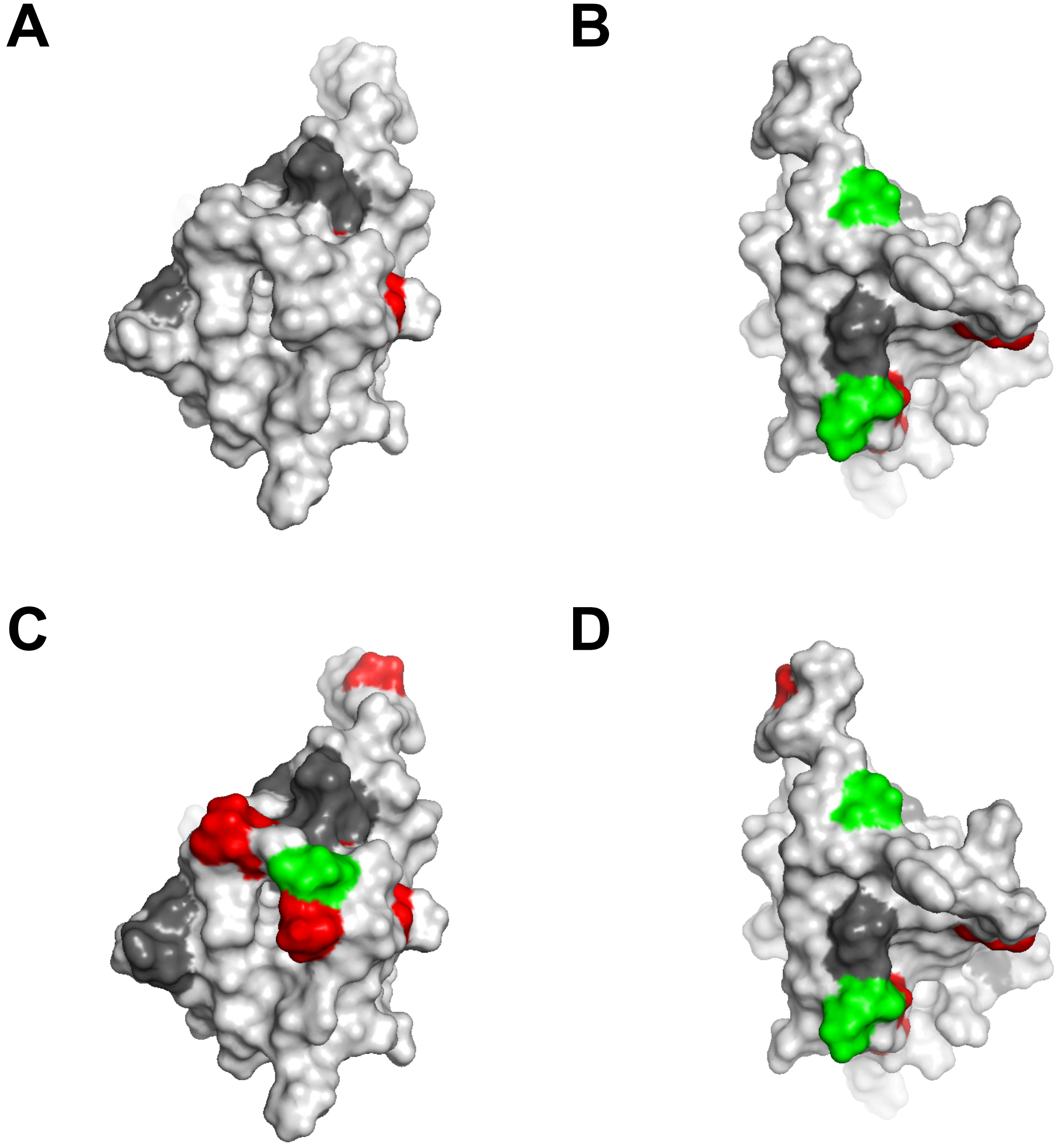

Figure S1. Surface plots of activities of synonymous variants in Tat. Surface maps show Tat structure (PDBID: 1TIV) with a 180° rotation. **A,B**: Stringent selection for variant activities that have a majority of replicate measures in different variants for the same position or in different cell lines. C,D: Non-stringent selection for variant activities that have a majority of replicate measures in different variants for the same position or in different cell lines, but includes variants that only have a single measure. Key: grey = wild type activity; green = GOF activity; and red = LOF activity
